# Psychological Backgrounds of Medically Compromised Patients and Its Implication in Dentistry: A Narrative Review

**DOI:** 10.3390/ijerph18168792

**Published:** 2021-08-20

**Authors:** Yoshihiro Abiko, Durga Paudel, Hirofumi Matsuoka, Mitsuru Moriya, Akira Toyofuku

**Affiliations:** 1 Division of Oral Medicine and Pathology, Department of Human Biology and Pathophysiology, School of Dentistry, Health Sciences University of Hokkaido, Hokkaido 061-0293, Japan; durga@hoku-iryo-u.ac.jp; 2 Division of Disease Control and Molecular Epidemiology, Department of Oral Growth and Development, School of Dentistry, Health Sciences University of Hokkaido, Hokkaido 061-0293, Japan; mazun@hoku-iryo-u.ac.jp; 3 Department of Psychosomatic Internal Medicine, Health Sciences University of Hokkaido Hospital, Hokkaido 002-8072, Japan; mmoriya@hoku-iryo-u.ac.jp; 4 Department of Psychosomatic Dentistry, Graduate School of Medical and Dental Sciences, Tokyo Medical and Dental University, Tokyo 113-8510, Japan; toyoompm@tmd.ac.jp

**Keywords:** anxiety, dental, depression, medically compromised patients, psychology

## Abstract

The number of medically compromised dental patients is increasing every year with the increase in the super-aged population. Many of these patients have underlying psychiatric problems and diseases, which need to be recognized by dental professionals for better treatment outcomes. The aim of this narrative review article is to summarize the psychological and psychiatric backgrounds of medically compromised patients who are frequently visited and taken care of by dentists using findings from recent systematic reviews and meta-analyses. Anxiety and symptoms of depression, post-traumatic stress disorders, panic disorders, poor cognitive functions, and poor quality of life were some of the common psychological backgrounds in medically compromised patients. Additionally, the consequences of these psychological problems and the considerations that need to be taken by the dentist while treating these patients have been discussed. Dental professionals should be aware of and recognize the different psychological backgrounds of medically compromised dental patients in order to provide appropriate dental treatment and to prevent oral conditions from worsening.

## 1. Introduction

The number of medically compromised patients is growing with the advance in the super-aged society in developed countries [1]. These patients need to be visited by a dentist more often with each progressing year. There are well established guidelines for how dentists see medically compromised patients for dental treatment [2]. Medically compromised patients may require regular prescriptions for their diseases or may experience an event that can interfere with their daily life. Many of these patients often have underlying psychological problems [3,4,5,6,7,8,9,10]. Dentists generally focus on dental problems, and the psychological backgrounds of these patients might often be neglected. The patient’s motivation and their self-efficacy, which can be affected by their psychology, are important for the prevention and treatment of dental diseases.

The aim of this review is to summarize the psychological backgrounds of commonly encountered medically compromised conditions in dental patients. Previous studies have shown that cardiovascular diseases such as hypertension and heart disease as well as diabetes are among the five most prevalent medically compromised conditions in dental patients [11]. Renal diseases have high global prevalence, occurring in nearly 10% of the population [12]. Additionally, connective tissue disease has a prevalence ranging from 5–30% after 65 years of age [13]. Based on these findings and our clinical experience, we selected four medically compromised conditions; diabetes, cardiovascular disease, renal disease, and connective tissue disease and reviewed the psychological status of the patients. Various psychological backgrounds in these diseases are discussed with reference to findings from recent systematic reviews and meta-analyses. The consequences of psychological status and the considerations that need to be taken by the dentist while treating these patients have also been discussed in this review.

## 2. Materials and Methods

An electronic search was made on PubMed using the following search terms for each of four diseases: (diabetes) AND (psychology), (cardiovascular disease) AND (psychology), (renal disease) AND (psychology), (connective tissue disease) AND (psychology). Articles in English and that had been published within the last 5 years were included (March 2015–March 2020). The studies were limited to systematic reviews and meta-analyses (Details in Supplementary Materials File S1). These articles formed the basis to show the psychological status of medically compromised patients. A few manually searched articles were also included in the review to discuss the implications of psychological background in dental treatment.

## 3. Results and Discussion

### 3.1. Diabetes

Diabetes is one of the four main non-communicable diseases (cardiovascular diseases, cancer, chronic respiratory diseases, and diabetes) and has a global prevalence of 8.5% among adults aged 18 years of age and older [14]. Some of the oral manifestations of diabetes include periodontal diseases, dental caries, candidiasis, burning sensation, altered taste, glossitis, and dry mouth [15]. The relationship between periodontal disease and diabetes is known to be bidirectional [16]. Diabetic patients have a high risk of periodontal diseases; the treatment of periodontal diseases in these patients may lead to a decrease in their blood glucose level [16].

Psychological approaches are often used in diabetic patients because they often suffer from psychological problems [17]. The psychological problems in diabetic patients might be due to diabetic distress [18], which is an emotional response to the burdens of living with diabetes and the self-care necessary to manage the condition. Diabetes-related distress has been reported in 36% of patients with type 2 diabetes [3]. Table 1 illustrates various psychological backgrounds of patients with diabetes [3,4,18,19,20,21,22,23,24,25,26,27,28,29,30,31]. The major psychological condition in diabetes includes depression and anxiety symptoms [4,26]. The pooled prevalence of depressive and anxiety symptoms in type 1 diabetic youth was shown to be 30% and 32%, respectively [32]. A large cohort study showed that the prevalence of major depression in diabetic patients was twice that in healthy individuals [33]. Moreover, diabetic patients showed poor cognitive performance [19,20,21,31] and may have a higher risk of dementia [34]. Severe hypoglycemia in type 2 diabetes was associated with increased fear of hypoglycemia, decreased emotional well-being and health status, and a diabetes-specific quality of life [35,36].

These psychological and psychiatric problems may complicate diabetic-related cardiovascular diseases, blood glucose level, and may worsen the quality of life [37]. Depression in patients with diabetes has been associated with increased risks for micro- and macrovascular complications and neuropathy [29,38]. Microvascular complications include retinopathy, neuropathy, and diabetic foot, whereas macrovascular complications include stroke, angina, cardiovascular diseases, and myocardial infarction. Furthermore, anxiety and stress may worsen the diabetic condition in these patients.

The effect of different types of psychological and psychiatric problems, including depressive conditions, may be seen during oral health and treatment. Depressive conditions in diabetes often decrease self-efficacy and the capacity for self-management [39], which might also lead to poor oral hygiene. Moreover, poor cognitive functions such as poor attention, slow psychomotor function, and poor processing speed in patients with diabetes compared to healthy individuals should be taken into consideration while planning dental treatment [19,31]. The increased risk of microvascular neuropathy in diabetic patients with depression should also be considered in dental treatment. Thus, dental professionals may need to recognize the psychological and psychiatric backgrounds of each diabetic patient in the dental setting.

### 3.2. Cardiovascular Diseases

Cardiovascular diseases are the number one cause of global death, constituting nearly one third of all global deaths [40]. Cardiovascular diseases include coronary heart disease, cerebrovascular disease, peripheral arterial disease, rheumatic heart disease, congenital heart disease, deep vein thrombosis, and pulmonary embolism. Acute events such as heart attack and stroke resulting from one or more cardiovascular diseases account for the majority of deaths and disabilities [40]. Dental professionals are generally cautious about underlying cardiovascular diseases during dental treatment. These patients need special care, mainly during oral surgical procedures and during medication administration. Blood pressure maintenance, hemorrhage prevention, and effects due to use of anticoagulants or anti-thrombin medications are often challenging during dental treatment in these patients [41].

Stress is a major exacerbation factor for hypertension, cardiac diseases, and arteriosclerosis [42,43]. Table 2 shows the psychological backgrounds of patients with cardiovascular diseases [5,6,42,43,44,45,46,47,48,49,50,51,52,53]. Anxiety and depression are major psychological factors in patients with cardiovascular diseases [5,48,49]. Furthermore, patients with cardiovascular diseases often have post-traumatic stress disorder (PTSD) induced by the event of a heart attack [6,50] and may experience anticipatory anxiety, which could be linked to other types of anxiety and depression. A recent meta-analysis showed a random-effects pooled prevalence of 13.1% for anxiety disorders, 28.79% for probable clinically significant anxiety, and 55.5% for elevated symptoms of anxiety among patients with heart failure [48]. The prevalence of depressive symptoms in males and females with coronary heart diseases was 23.47% and 35.75%, respectively, at the time of hospital admission [54]. The risk of developing depression in patients with coronary artery disease was 2.8 times higher than that in healthy individuals [55]. Patients with ischemic heart disease must practice self-care methods to prevent the occurrence of events, which can become a new stressor. These stresses in combination with anticipatory anxiety may be responsible for the high prevalence of anxiety and depression in patients with cardiovascular diseases.

Both anxiety and depression are associated with an increased risk of mortality in patients with coronary artery diseases [53]. Depression is an independent predictor of all-cause mortality and might be a marker of severe heart failure [44,56]. Depression is also closely related to hypertension; depressive symptoms have been known to induce hypertension [57], and about 40.1% of hypertensive patients are reported to suffer from depression [58]. Hypertensive patients with depression have poor health status and a lower quality of life and demonstrate decreased treatment compliance [45]. Several review articles have indicated that cardiovascular diseases often induce psychological problems. Patients with arrhythmia frequently experience anxiety, whereas patients with paroxysmal atrial fibrillation often present with avoidant behavior due to fear of paroxysm [59]. Furthermore, patients with implantable cardiac defibrillators are fearful of electric shock [60].

Patients with cardiovascular diseases often experience several levels of anxiety and depression. Those suffering from anxiety and depression often have less distress tolerance, which may cause dental phobia [61]. Furthermore, stressful conditions such as dental treatments may worsen their psychological problems. Depression in cardiovascular disease is associated with decreased treatment compliance and non-adherence to medication, which may affect the prognosis of dental treatment [45,46]. Poor cognitive outcomes such as non-verbal reasoning, processing speed and attention, psychomotor abilities, and numeracy in patients with cardiovascular diseases may require consideration in dental treatment [47,62].

### 3.3. Renal Diseases

Renal diseases are among significant contributors to morbidity and mortality from non-communicable diseases globally [63]. Nation surveys in Australia, Norway, and the USA showed that at least 10% of the adult population have markers for kidney disease. A global prevalence of 9.1% has been shown for cases of all-stage chronic kidney diseases (CKD) [12]. Dental professionals commonly encounter renal patients during dental treatment and should be cautious of their tendency for hemorrhage and the increased susceptibility to infection in dialysis patients [64,65]. Additionally, certain drugs involved in renal metabolism have to be carefully prescribed for such patients [65].

CKD is characterized by a gradual loss of kidney function requiring frequent dialysis, which impairs the activities of daily living. Dialysis patients are at a significant increased risk for sarcopenia and frailty [66] and are under constant psychological stress. Many of these patients experience psychological and psychiatric problems, and the study of the psychological factors in these patients has been termed as “psychonephrology” [67]. The psychological backgrounds of patients with renal diseases are shown in Table 3 [7,8,68,69,70,71,72,73,74,75]. Dialysis patients without any psychiatric history often develop sleep disturbances, depression, anxiety, delirium, dementia, and restless leg syndrome [67]. Depression was reported as one of the most prevalent mental diseases in hemodialysis patients, and the depressive symptoms were found to be closely associated with malnutrition [7]. A previous study showed a pooled prevalence of 62% for depressive symptoms in patients undergoing hemodialysis [68]. The depressive symptoms in CKD patients might be due to these negative emotions and distress [69] as well as the malnutrition in those undergoing hemodialysis. Physical disorders such as uremia, hypercalcemia, and cerebrovascular damage, along with psychological stress, can also cause depressive symptom [76]. Furthermore, medications such as steroids, interferon, antihypertensive drugs, and anti-parkinsonism drugs can induce depression in CKD patients [77].

The depression symptoms in dialysis patients may demonstrate decreased adherence to treatment, which affects oral health care. Studies have shown that social support from family and health care professionals is associated with treatment adherence in CKD patients [75]. The treatment adherence of patient is an important factor in successful dental treatment. Dialysis patients are at increased risk of developing dementia [67], which may present with an increased risk for physical frailty, including oral frailty [78]. CKD has a significant impact on cognition, which might diminish the ability of the patient to make health care decisions [72]; this factor should be considered during dental treatment. Thus, dental professionals must be aware of the psychological and psychiatric background of patients with renal diseases, particularly those undergoing dialysis, in a dental setting.

### 3.4. Connective Tissue Diseases

Connective tissue diseases refer to a group of heterogenous and immunologically mediated disorders such as rheumatoid arthritis, systemic lupus erythematosus, systemic sclerosis, and Sjogren’s syndrome. The prevalence ranges from 5–30% after the age of 65 years [13]. Oral manifestations may appear during the early phases of these diseases, and dental professionals may need to identify, manage, and refer accordingly.

The oral symptoms in connective tissue diseases mainly manifest as xerostomia and aphthous stomatitis. Those patients with connective tissue diseases and who are taking steroids have an increased susceptibility to infections and are at a high risk for the progression of periodontal diseases and opportunistic infections such as candidiasis [79,80]. Moreover, patients with hyposalivation are prone to oral infections [81]. Chronic inflammation in patients with connective tissue diseases induces fatigue and stress, and these diseases may be involved in the development of psychiatric diseases caused by neuritis [82].

The psychological backgrounds of patients with connective tissue diseases are shown in Table 4 [9,83,84,85,86,87,88,89,90,91]. Fatigue associated with psychosocial factors, such as depression, pain, and sleep disorders, were the most prevalent symptom in patients with connective tissue diseases [84]. Each type of connective tissue disease is associated with a different type of psychological problem. Rheumatoid arthritis patients experience fatigue [84,86] and poor cognitive function [90] and need to be treated for issues such as negative emotions, anxiety, and depression. Therapeutic interventions for immunological disorders improve both depression and the pathological condition in patients with rheumatoid arthritis, indicating that immunological disorders may be involved in causing depression in these patients [92]. Patients with systemic lupus erythematosus (SLE) frequently present with various types of psychiatric problems, such as impaired consciousness, anxiety disorders, cognitive disorders, mood disorders, and schizophrenia-like psychosis, which are collectively termed as neuropsychiatric SLE (NPSLE) [93,94]. Sjögren’s syndrome, a collagen-related disease, affects the senses of smell and taste, sexual function, and quality of life [88]; Approximately 33.8% and 36.9% of patients with this syndrome were reported to experience anxiety and depression, respectively [95]. The odds ratio of depression in patients with Sjögren’s syndrome, when compared to that of healthy individuals, was 5.32 [95]. Patients with connective tissue diseases who take steroid medications often have mood disorders and hallucinations, which is termed as “steroid psychosis” [96]. A high dose of steroids frequently induces this condition in patients, making it difficult for the clinician to ascertain the cause of the symptoms [97].

Patients with connective tissue diseases may have complex psychiatric problems because both immunological disorders and psychological stress can induce psychiatric diseases. Patients with connective tissue diseases often experience fatigue, which is associated with psychosocial factors such as pain, depression, sleep disorders, and low mood [84,85,86]. These factors should be considered during dental treatment. Previous studies have shown a significant association between the oropharyngeal manifestations of systemic sclerosis (assessed as maximal mouth opening and mouth handicap in systemic sclerosis scale) and impaired quality of life [89]. These factors are particularly of a dentist’s concern and should be considered during the dental treatment of these patients.

## 4. Psychology and Its Implication in Dentistry

The effect of various psychological backgrounds of patients with diabetes, cardiovascular diseases, renal diseases, and connective tissue diseases in dental treatment have been discussed in this article. The most common psychological states in those patients were anxiety, depression, post-traumatic stress disorders, panic disorders, poor cognitive functions, and poor quality of life. In general, psychological stress, anxiety, and depression have been linked to many oral mucosal diseases, such as burning mouth syndrome, lichen planus, and the recurrence aphthous stomatitis [98,99,100]. Psychological stresses have also been associated with periodontal diseases [101] and dental caries [102]. Negative human behavior such as low self-efficacy in adverse psychological conditions might worsen the oral environment [103]. A systematic review and meta-analysis studying the effect of psychological state on oral health showed that all of the psychiatric diagnoses were associated with increased dental caries and tooth loss on the mean number of decayed, missing, and filled teeth (DMFT) or surfaces (DMFS) scores (OR = 1.22; 95%CI = 1.14–1.30) [104]. The psychological states of medically compromised patients could therefore affect the oral health. The periodic monitoring of oral health and needful intervention at earlier stages of oral disease might be important.

The limitation of our study is that it is a narrative review and strict study selection criteria were not followed. The results of systematic reviews and meta-analyses were cited to discuss the possible psychological backgrounds of medically compromised patients. The studies highlighting the psychological status of dental patients with medically compromised conditions are very limited, and this topic needs to be further studied.

## 5. Conclusions

This review focused on the psychological and psychiatric backgrounds of medically compromised dental patients, i.e., those with diabetes, cardiovascular disease, renal disease, and connective tissue diseases. The psychological backgrounds of medically compromised patient are diverse. Dental professionals should be aware of and recognize these backgrounds in addition to the systemic conditions in order to provide the appropriate dental treatment and to prevent the oral conditions from worsening.

## Figures and Tables

**Table 1:** Psychological background of patients with diabetes.

		References
***A.***	***Poor quality of life***	
	Depression, anxiety, and worry in diabetes is associated with poor quality of life [OR = 3.0 (95% CI: 1.135–7.948)]	[19]
***B.***	***Diabetes as a risk factor for psychological problems***	
	Patients with diabetes have an increased risk of developing anxiety (OR = 1.48 (95% CI: 1.27–1.74)) and depressive symptoms (RR = 1.29 (95% CI: 1.03–1.63))	[4,26]
	Diabetic distress is present in about one-third of type 1 and type 2 diabetes mellitus patients	[3,18]
	Depression, anxiety, low quality of life, and poor sleep are associated with painful diabetic neuropathy	[21]
***C.***	***Diabetes with depression leading to other complications***	
	Patients with diabetes and depression have poorer cognitive function compared to those with diabetes alone	[22]
	Patients with diabetes and depression have an increased risk of microvascular (retinopathy, neuropathy) ((HR = 1.38 (95% CI: 1.30–1.47)) and macrovascular complications (stroke, angina, cardiovascular diseases) ((HR = 1.33 (95% CI: 1.25–1.41))	[23]
***D.***	***Diabetes as a risk factor for suicide***	
	Patients with diabetes have an increased risk of suicide (RR = 1.56; (95% CI: 1.29–1.89))	[24]
***E.***	***Poor cognition***	
	Diabetes in children and adults is associated with cognitive dysfunction such as lowered intelligence, diminished attention, and slowing of psychomotor speed	[20,25,27,28]
***F.***	***Anxiety/Stress as a risk factor for diabetes***	
	Anxiety might be a risk factor for diabetes (OR = 1.47 (95% CI: 1.23–1.75))	[29]
	Women are associated with work-related stress and risk of diabetes (RR = 1.22 (95% CI: 1.01–1.46))	[30]
	Workplace bullying victims had 1.46 times higher risk of developing diabetes compared to those who had not experienced workplace bullying (HR = 1.46 (95% CI: 1.23–1.74))	[31]

**Table 2:** Psychological backgrounds of patients with cardiovascular diseases.

		References
***A.***	***Anxiety and depression in cardiovascular patients***	
	Prevalence of anxiety in heart failure: anxiety disorders (13.1%), clinically significant anxiety (28.7%), and elevated symptoms of anxiety (55.5%) Prevalence of depression in peripheral arterial disease: 3% to 48% prevalence of depression in myocardial infarction: female (36%), male (29%)	[5,44,45]
***B.***	***Post-traumatic Stress Disorder (PTSD)***	
	Prevalence of cardiac disease-induced PTSD averaged 12%	[46]
***C.***	***Panic Disorders***	
	Panic disorder is associated with incident coronary heart disease (HR = 1.47 (95% CI: 1.25–1.74)) and myocardial infarction (HR = 1.36 (95% CI: 1.45–1.85))	[47]
***D.***	***Loneliness and social isolation***	
	Poor social relationships are associated with a 29% increase in the risk of incident coronary heart disease	[48]
***E.***	***Anxiety and depression as a risk of mortality***	
	Anxiety and depression are associated with an increased risk of mortality in patients with coronary artery disease (OR = 1.21 (95% CI:1.06–1.39)) and heart failure (HR = 1.57 (95 %CI: 1.30–1.89)), respectively	[49,50]
***F.***	***Cardiovascular diseases and depression leading to decreased treatment compliance***	
	Depression in hypertensive patients and acute coronary syndrome patients associated with decreased treatment compliance (Prevalence-26.8%) and non-adherence to medication (OR = 2.00 (95% CI: 1.57–3.33)), respectively	[51,52]
***G.***	***Poor cognition***	
	Prevalence of cognitive impairment in heart failure patients averaged 43% (OR = 1.67 (95% CI: 1.15–2.42))	[53]
***H.***	***Stress/depression symptoms as a risk factor for cardiovascular diseases***	
	Psychosocial stress such as occupational stress, socioeconomic status, anxiety, and depression are associated with an increased risk of hypertension (OR = 2.40 (95% CI = 1.65–3.49))	[42]
	Depressive symptoms contributed to subclinical atherosclerosis with impaired functional and structural markers	[43]

**Table 3:** Psychological backgrounds of patients with renal diseases.

		References
***A.***	***Anxiety, Distress, and Depression***	
	Depression is one of the most prevalent mental illnesses (ranging from 6–83.5% among studies) in hemodialysis patients.	[7,68]
	Chronic Kidney Disease (CKD) patients have negative emotions and distress. Disease severity and illness perception is associated with depression	[69]
	Low albumin, high Interlukin-6, and high C-Reactive Protein are associated with the severity of depressive symptoms in chronic renal disease	[70]
	Kidney transplant patients are exposed to a high risk of psychiatric disorders such as mental distress, behavioral and adaptation difficulties, cognitive impairments and depressive symptoms, sleep disorders, anxiety, and depression	[71]
***B.***	***Post-Traumatic Stress Disorder (PTSD)***	
	PTSD is common in patients with solid organ transplants, including renal transplant	[8]
***C.***	***Effect on cognition***	
	Renal patients experienceeffects on orientation and attention, language, concept formation and reasoning, executive function, memory, and global cognition. The cognitive impact might diminish the patient’s ability to make health care decisions	[72]
	The pooled prevalence of cognitive impairment among peritoneal dialysis patients is 28.7%	[73]
***D.***	***Effect on quality of life***	
	Renal failure is associated with lower quality of life in young adults	[74]
***E.***	***Need for social support***	
	A significant association between social support and treatment adherence is seen in patients with end-stage renal disease.	[75]

**Table 4:** Psychological backgrounds of patients with connective tissue diseases.

		References
***A.***	***Anxiety and depression***	
	The depressive (6.7% to 59%) and anxiety symptoms (34% to 37%) are prevalent in childhood-onset systemic lupus erythematosus (SLE)	[9]
	Patients with rheumatoid arthritis are associated with an increased risk of anxiety OR = 1.20 (95% CI: 1.03–1.39)	[83]
***B.***	***Fatigue in connective tissue diseases and its psychosocial factors***	
	Fatigue in SLE is associated with psychosocial factors (depression, pain, and sleep disorders)	[84]
	The dominant unpredictability of rheumatoid arthritis-related fatigue is experienced as a vicious circle described in relation to its physical, cognitive, emotional, and social impact	[85]
	Low mood is associated with increased fatigue in rheumatoid arthritis patients	[86]
***C.***	***Poor quality of life***	
	SLE has a significant impact on the health-related quality of life (Mental component summary = 50.37 (95% CI: 47.78–52.87))	[87]
	Primary Sjögren’s syndrome has an impact on quality of life in female patients (mental component of the quality of life = −0.83 (95% CI: −1.27 to −0.40))	[88]
	There is a significant association between the oropharyngeal manifestations of systemic sclerosis (assessed as maximal mouth opening and mouth handicap in systemic sclerosis scale) and impaired quality of life	[89]
***D.***	***Poor cognitive function***	
	A significant underperformance in cognitive function tests, mainly verbal functions, memory, and attention in rheumatoid arthritis patients is observed when compared to controls	[90]
***E.***	***Suicide ideation and attempt***	
	Rheumatic diseases patients have a high pooled prevalence of suicidal ideation (26%) and suicide attempts (12%). The prevalence of suicidal ideation and a suicidal attempts is higher in females than in males	[91]

## Data Availability

Not applicable.

## Supplementary Materials

The following are available online at https://www.mdpi.com/article/10.3390/ijerph18168792/s1, File S1: Search strategy.
