# Supplementary material for: Psychological Backgrounds of Medically Compromised Patients and Its Implication in Dentistry: A Narrative Review
Source: Int J Environ Res Public Health. 2021 Aug 20;18(16):8792. doi: 10.3390/ijerph18168792 (PMC8392062; doi:10.3390/ijerph18168792)
Supplement: Supplementary file 1 [file ijerph-18-08792-s001.zip › ijerph-1307390-supplementary.pdf]

Supplementary File S1
Search strategy
An electronic search was made on PubMed using the following search terms for each of four diseases: (diabetes) AND (psychology), (cardiovascular disease) AND (psychology), (renal disease) AND (psychology), (connective tissue disease) AND (psychology).
The search builder used following queries to search for each disease.

- ("diabete"[All Fields] OR "diabetes mellitus"[MeSH Terms] OR ("diabetes"[All Fields] AND "mellitus"[All Fields]) OR "diabetes mellitus"[All Fields] OR "diabetes"[All Fields] OR "diabetes insipidus"[MeSH Terms] OR ("diabetes"[All Fields] AND "insipidus"[All Fields]) OR "diabetes insipidus"[All Fields] OR "diabetic"[All Fields] OR "diabetics"[All Fields] OR "diabets"[All Fields]) AND ("psychologie"[All Fields] OR "psychologies"[All Fields] OR "psychology"[MeSH Subheading] OR "psychology"[All Fields] OR "psychology"[MeSH Terms] OR "psychology s"[All Fields])

- ("cardiovascular diseases"[MeSH Terms] OR ("cardiovascular"[All Fields] AND "diseases"[All Fields]) OR "cardiovascular diseases"[All Fields] OR ("cardiovascular"[All Fields] AND "disease"[All Fields]) OR "cardiovascular disease"[All Fields]) AND ("psychologie"[All Fields] OR "psychologies"[All Fields] OR "psychology"[MeSH Subheading] OR "psychology"[All Fields] OR "psychology"[MeSH Terms] OR "psychology s"[All Fields])

- ("kidney diseases"[MeSH Terms] OR ("kidney"[All Fields] AND "diseases"[All Fields]) OR "kidney diseases"[All Fields] OR ("renal"[All Fields] AND "disease"[All Fields]) OR "renal disease"[All Fields]) AND ("psychologie"[All Fields] OR "psychologies"[All Fields] OR "psychology"[MeSH Subheading] OR "psychology"[All Fields] OR "psychology"[MeSH Terms] OR "psychology s"[All Fields])

- ("connective tissue diseases"[MeSH Terms] OR ("connective"[All Fields] AND "tissue"[All Fields] AND "diseases"[All Fields]) OR "connective tissue diseases"[All Fields] OR ("connective"[All Fields] AND "tissue"[All Fields] AND "disease"[All Fields]) OR "connective tissue disease"[All Fields]) AND ("psychologie"[All Fields] OR "psychologies"[All Fields] OR "psychology"[MeSH Subheading] OR "psychology"[All Fields] OR "psychology"[MeSH Terms] OR "psychology s"[All Fields])

The filters applied were: Timeline set to year 2020; articles in the last 5 years; studies limited to systematic review and meta-analysis; articles in English.
After applying filters, resulting studies were further screened for their final inclusion.

	Disease	Search term	Search result (number of articles)	After applying filters (number of articles)	Final inclusion (number of articles)
1	Diabetes	(diabetes) AND (psychology)	28317	437	16
2	Cardiovascular disease	(cardiovascular disease) AND (psychology)	61281	942	13
3	Renal disease	(renal disease) AND (psychology)	8474	111	10
4	Connective tissue disease	(connective tissue disease) AND (psychology)	7200	101	10
